# Orphan Genes in Crop Improvement: Enhancing Potato Tuber Protein without Impacting Yield

**DOI:** 10.3390/plants11223076

**Published:** 2022-11-13

**Authors:** Rezwan Tanvir, Lei Wang, Amy Zhang, Ling Li

**Affiliations:** 1 Department of Biological Sciences, Mississippi State University, Starkville, MS 39762, USA; 2 Mississippi School for Mathematics and Science, Columbus, MS 39701, USA

**Keywords:** *QQS*, orphan gene, *NF-YC4*, *Solanum tuberosum*, carbon and nitrogen allocation, tuber yield, tuber starch, tuber protein

## Abstract

*Qua-Quine Starch* (*QQS*), an *Arabidopsis thaliana* orphan gene, and its interactor, Arabidopsis Nuclear Factor Y subunit C4 (AtNF-YC4), can increase the total leaf and seed protein in different plants. Despite their potential in developing protein-rich crop varieties, their influence on the protein content of the stem, modified stem, and tuber was never investigated. Potato (*Solanum tuberosum*) is one of the most valuable food crops worldwide. This staple food is rich in starch, vitamins (B_6_, C), phenolics, flavonoids, polyamines, carotenoids, and various minerals but lacks adequate proteins necessary for a healthy human diet. Here we expressed *A. thaliana QQS* (*AtQQS*) and overexpressed *S. tuberosum NF-YC4* (*StNF-YC4*) in potatoes to determine their influence on the composition and morphological characteristics of potato tubers. Our data demonstrated higher protein and reduced starch content in potato tubers without significantly compromising the tuber yield, shape, and numbers, when *QQS* was expressed or *StNF-YC4* was overexpressed. Publicly available expression data, promoter region, and protein–protein interaction analyses of *StNF-YC4* suggest its potential functionality in potato storage protein, metabolism, stress resistance, and defense against pests and pathogens. The overall outcomes of this study support *QQS* and *NF-YC4*’s potential utilization as tools to enhance tuber protein content in plants.

## 1. Introduction

Potato, the fourth most important crop consumed, is considered a staple food that feeds millions of people worldwide [1]. Potato tubers synthesize and store starch and generally have a high starch content [2]. Due to the presence of excessive starch, potatoes are palatable and have several other agricultural and nutritional merits that make them popular in different parts of the world [1,3]. Despite its widespread use as a staple food, the crop usually offers inadequate protein to its consumers and poses a malnutrition threat to populations that heavily rely on potatoes as the primary source of nourishment [4]. The popularity of plant-based proteins for consumption has surged in recent years as they offer higher sustainability and affordability [5,6]. Plant proteins are also well-known for their health benefits. They are associated with lowered mortality rates, especially lowered cardiovascular mortality, reduced risk from chronic kidney disease, reduced resistance to insulin, and reduced obesity while providing all necessary essential amino acids [5,7,8,9,10]. High-protein potatoes can help combat world hunger, malnutrition, and ever-increasing protein deficiency [1,11]. Recent agricultural revolutions have delivered many high-yielding nutritious varieties of different crops, but suitable protein-rich potato varieties are still scarce.

*Qua-Quine Starch* (*QQS*) is a small gene exclusively found in *Arabidopsis thaliana* (no known homolog in any other species) and is considered an *A. thaliana* species-specific orphan gene [12,13]. It interacts with the Nuclear Factor Y subunit C4 (NF-YC4) [14]. *QQS* regulates carbon and nitrogen allocation, increases protein, decreases starch, and can increase plant pest and pathogen resistance in different plant species [13,14,15,16,17,18,19]. NF-YC4, a component of the NF-Y transcription factor conserved among all eukaryotes, can also regulate carbon and nitrogen allocation on its own without *QQS* to enhance total protein and pest and pathogen resistance in plants [14,16,17,19]. Furthermore, NF-Y is also reported to be involved in organisms’ growth, development, reproduction, hormone signaling, and biotic and abiotic stress response [14,19,20,21,22,23,24,25,26].

Several examples of plant orphan genes have been reported to play an important role in organisms’ metabolism, homeostasis, growth, development, reproduction, and different biotic–abiotic stress responses [12,13,27,28,29,30,31,32,33,34,35,36]. Surprisingly, some reports confirmed orphan genes could perform their functions even in nonnative plant species where no homologs or motifs of these unique genes are present [14,15,27,36]. For example, *A. thaliana*-specific *AtQQS* can function in soybean, rice, corn, and tobacco, where it increases the protein content in the leaf and seed of the plant [14,15,16,17]. QQS interactor NF-YC4 was reported to increase protein content independently of QQS in leaves and seeds of Arabidopsis, soybean, corn, and tobacco [14,16,17,18]. Recent studies reported that *QQS* and *NF-YC4* conferred enhanced pest and pathogen resistance in soybean and tobacco [17,19]. However, it remains elusive whether *QQS* and *NF-YC4* could extend their functionality to crops to elevate protein contents in stems, modified stems, or tubers.

To explore the effect of *QQS* and *NF-YC4* in plant tubers, *AtQQS* was introduced in the Atlantic (ATL) variety (*AtQQS-E*), and *StNF-YC4* (AtNF-YC4 homolog in potato, PGSC0003DMT400039470 in International Potato Genome Sequencing Consortium or PGSC database, Supplementary Table S1) was overexpressed in the Clearwater (CWR) variety (*StNF-YC4-OE*) of potato (*Solanum tuberosum*), respectively. We hypothesized that *QQS* and *NF-YC4* may interact with metabolic networks in potato tubers and increase the protein content in tubers. Protein quantification showed an increase in total protein content, and starch quantification showed a decrease in starch content in *AtQQS-E* and *StNF-YC4-OE* tubers. Sodium Dodecyl Sulphate–Polyacrylamide Gel Electrophoresis (SDS-PAGE) did not detect an increase of any specific protein in the mutants. *QQS* expression and *NF-YC4* overexpression can increase total tuber protein in potatoes. RNA-Seq data analysis revealed that *StNF-YC4* is highly expressed in all parts of the potato plant. Promoter region analysis of *StNF-YC4* identified several transcription factor binding sites and *cis*-acting elements associated with metabolism, storage protein, stresses, pathogen, and pest resistance. Our research demonstrates the ectopic expression of an orphan gene *QQS*, and overexpression of its interactor *NF-YC4* can alter the composition of plant stems and tubers, implicating the immense potential of orphan genes in crop development and biotechnology.

## 2. Results

### 2.1. No Significant Difference Was Observed in Tuber Yield, Number, and Appearance in AtQQS-E and StNF-YC4-OE Potato Plants vs. Wild-Type Control

**Figure 1:**
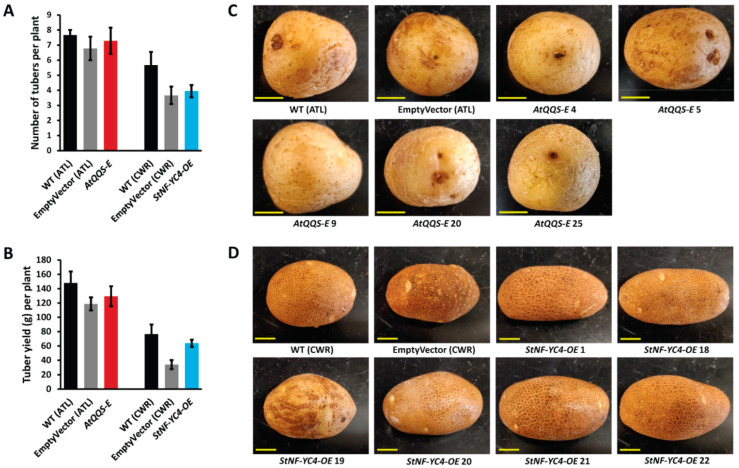
Tuber number, tuber yield, and tuber appearance were not impacted by *AtQQS* expression and *StNF-YC4* overexpression. Number of potato tubers (**A**) and tuber yield (**B**) per plant, and the appearance of *AtQQS-E* (**C**) and *StNF-YC4-OE* (**D**) tubers. All tubers expressing *AtQQS*, WT (ATL), and EmptyVector (ATL) are of the Atlantic variety, and tubers overexpressing *StNF-YC4*, WT (CWR), and EmptyVector (CWR) are of the Clearwater variety. All data in the bar chart show mean ± SE (Standard Error), *n* = 3. Student’s *t*-test was used to compare *AtQQS-E*, *StNF-YC4-OE*, and corresponding WTs in (**A**,**B**). None of the changes in (**A**,**B**) were significant (*p* > 0.05). *AtQQS-E*, *StNF-YC4-OE* tubers had similar sizes, shapes, and appearances to the corresponding WT tubers in (**C**,**D**). Scale bar in (**C**,**D**), 1 cm.

Potato plants expressing *AtQQS* or overexpressing *StNF-YC4* (Supplementary Figure S1) had a slightly reduced number of tubers per plant compared to their wild-type (WT) counterparts (Figure 1A). The tuber yield per plant was also marginally decreased from the WT plants (Figure 1B). Similarly, tuber yield and number are slightly reduced in plant lines with empty vectors. However, none of these changes were significant (*p* > 0.05). All ATL variety of tubers were round and uniform, while CWR tubers were oblong to long. No remarkable visual changes in appearance were observed in the tubers of *AtQQS-E* and *StNF-YC4-OE* potato plants when compared to their WT counterparts (Figure 1C,D). Tubers with similar size, shape, and appearance were selected to perform all experiments in this study.

### 2.2. AtQQS and StNF-YC4 Were Expressed and Overexpressed in Transgenic Potato Tubers

**Figure 2:**
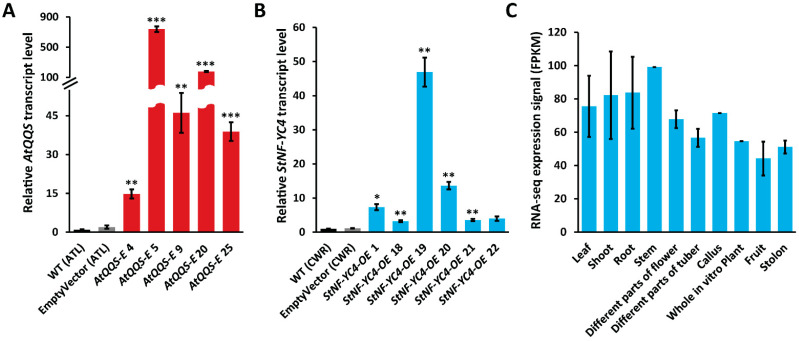
Expression of *AtQQS*, *StNF-YC4* in transgenic potato tubers and *StNF-YC4* expression in potato plant. Relative transcript levels of *AtQQS* (**A**) and *StNF-YC4* (**B**) in the tuber tissue of transgenic potatoes were quantified using the *EF-1α* gene as a reference by RT-qPCR. RNA-Seq expression signal (FPKM) of *StNF-YC4* (**C**) in different organs of potato plants. The data set was collected from publicly available online resources [37,38,39]. All data in the bar chart show mean ± SE, *n* = 3. All tubers in (**A**) are of ATL variety, and tubers in (**B**) are of CWR variety. Student’s *t*-test was used to compare *AtQQS-E*, *StNF-YC4-OE*, and corresponding WTs in (**A**,**B**), *** *p* < 0.001; ** *p* < 0.01; * *p* < 0.05.

To confirm the expression of *AtQQS* and overexpression of *StNF-YC4* in potato tubers, RT-qPCR was performed to determine their transcript level in *AtQQS-E* and *StNF-YC4-OE* mutants using mRNA extracted from tuber tissue. *AtQQS-E* lines had a high accumulation of *QQS* transcript ranging from 15- to 730-fold (Figure 2A, *p* < 0.01) compared to WT (ATL) and EmptyVector (ATL). *StNF-YC4-OE* had a significantly higher *StNF-YC4* transcript level between 3- to 47-fold compared to the WT (CWR) and EmptyVector (CWR) plants (Figure 2B, *p* < 0.05). Another independent RT-qPCR test with a different set of *AtQQS* primers and reference gene confirmed *AtQQS* expression in *AtQQS-E* potato leaves (Supplementary Figure S2).

### 2.3. StNF-YC4 Expression Is Universal in Different Organs of the Potato Plant

One set of RNA-Seq data from past studies [37,38,39] showed high *StNF-YC4* expression in different organs of the potato plant, including shoot apex (108.58 FPKM (Fragments Per Kilobase of exon per Million mapped fragments)), stem (99.11 FPKM), roots (105.28 FPKM), and flower petioles (129.69 FPKM), and moderate expression in tubers (~70 FPKM), leaves (56.61 FPKM), and different flower parts (~67 FPKM) (Figure 2C, Supplementary Figure S3). More specifically, the mature tuber had an expression signal FPKM of 69.01, while the tuber cortex and pith had 74.37 and 69.99, respectively (Supplementary Figures S3 and S4). Our RT-qPCR study also detected *StNF-YC4* transcript in potato tubers and confirmed its overexpression in *StNF-YC4-OE* tubers (Figure 2B).

### 2.4. Ectopic Expression of AtQQS and Overexpression of StNF-YC4 in Potatoes Reduces Starch Accumulation in the Tubers

**Figure 3:**
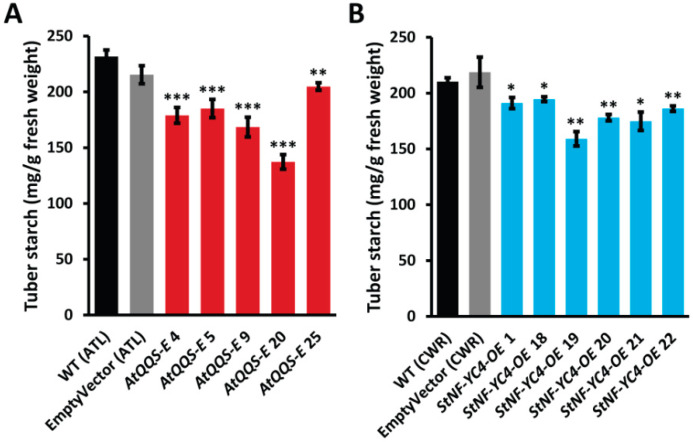
Starch accumulation was decreased in potato tubers expressing *AtQQS* and overexpressing *StNF-YC4-OE*. The starch content of *AtQQS-E* (**A**) and *StNF-YC4-OE* (**B**) potato tubers were quantified by Megazyme’s GOPOD starch quantification assay. All data in the bar chart show mean ± SE, *n* ≥ 4. All tubers in (**A**) are of ATL variety, and tubers in (**B**) are of CWR variety. Student’s *t*-test was used to compare starch contents of *AtQQS-E* and *StNF-YC4-OE* to corresponding WT, *** *p* < 0.001; ** *p* < 0.01; * *p* < 0.05.

Starch quantification data showed that *AtQQS-E* tubers had a significant decrease in starch content that ranged between 10–41% when compared to WT (ATL) (Figure 3A). All six lines of potato tubers overexpressing *StNF-YC4* had a 4–24% decrease in starch accumulation compared to the WT (CWR) tubers (Figure 3B). All decreases were significant, except tubers with an empty vector did not produce a significant change in tuber starch content compared to the WT. Our data revealed that both *QQS* and *NF-YC4* decreased starch content in the plant stem in potato tubers.

### 2.5. AtQQS-E and StNF-YC4-OE Potato Plants Have Higher Protein Accumulation in the Tubers

**Figure 4:**
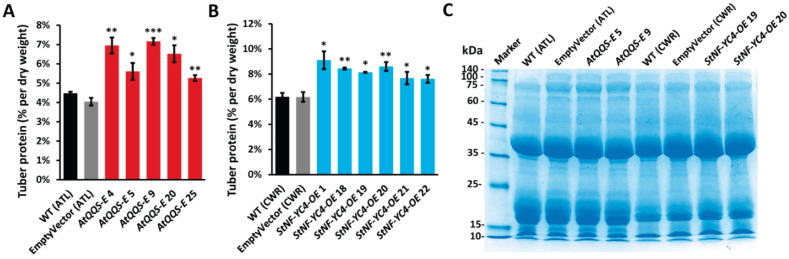
The total protein was uniformly increased in *AtQQS-E* and *StNF-YC4-OE* potato tubers. Modified Lowry protein assay was used to quantify total protein content in *AtQQS-E* (**A**) and *StNF-YC4-OE* (**B**) potato tubers. All data in bar charts (**A**,**B**) show mean ± SE, *n* ≥ 4. Student’s *t*-test was used to compare protein contents of *AtQQS-E*, *StNF-YC4-OE*, and corresponding WT in (**A**,**B**), *** *p* < 0.001; ** *p* < 0.01; * *p* < 0.05. (**C**) SDS-PAGE analysis of total protein in *AtQQS-E* and *StNF-YC4-OE* potato tubers. An equal amount of total protein extracted from potato tubers was used for each SDS-PAGE sample in (**C**). All tubers expressing *AtQQS*, WT (ATL), and EmptyVector (ATL) are of the ATL variety, and tubers overexpressing *StNF-YC4*, WT (CWR), and EmptyVector (CWR) are of the CWR variety.

To determine the total protein content, we performed protein quantification using the modified Lowry assay. We found that tubers expressing *AtQQS* had higher total protein content than the WT (ATL) and EmptyVector (ATL), ranging from a 15–60% increase (Figure 4A). Lines overexpressing *StNF-YC4* showed a 23–52% increase in total protein compared to the WT (CWR) and EmptyVector (CWR) tubers (Figure 4B). No specific protein (or group of proteins) increase was detected by SDS-PAGE in the mutants (Figure 4C).

### 2.6. Sequence Similarity to Other NF-YC4 Homologs Shows Multiple Conserved Regions among Them

**Figure 5:**
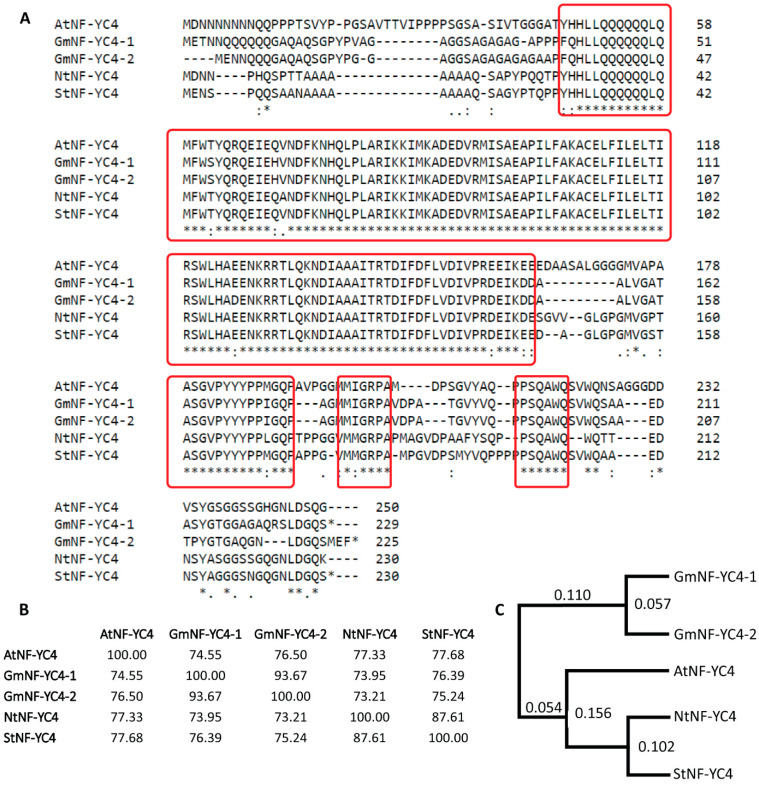
Sequence similarity of StNF-YC4 to other NF-YC4 homologs from different species. (**A**) Protein sequence alignment of StNF-YC4 with homologs in *A. thaliana* (AtNF-YC4), soybean (GmNF-YC4-1 and GmNF-YC4-2), and tobacco NtNF-YC4. Conserved sequences are outlined in red. The “*” at the end of the StNF-YC4, GmNF-YC4-1, and GmNF-YC4-2 sequences signify a stop codon. “*“ under the amino acid sequence indicates positions with a single, fully conserved residue, “:” indicates conservation between groups of strongly similar properties (scoring > 0.5 in the Gonnet PAM 250 matrix), and “.” indicates conservation between groups of weakly similar properties (roughly equivalent to scoring ≤ 0.5 and >0 in the Gonnet PAM 250 matrix). (**B**) A percent identity matrix shows similarities among the NF-YC4 proteins in *A. thaliana*, soybean, and tobacco peptide sequences. The matrix was created by Clustal 2.1 [47]. (**C**) A maximum-likelihood tree for the NF-YC proteins in *A. thaliana*, soybean, tobacco, and potato. A blastp search was performed using the AtNF-YC4 amino acid sequence as the query, and the top match from *S. tuberosum* was selected for the tree. Branch lengths were measured in the number of substitutions per site.

In past studies, we identified several conserved regions among different plant homologs of the NF-YC4 peptide sequence [16,17]. Using sequence alignment, we compared NF-YC4 homologs against StNF-YC4 (Figure 5A). Our results found identical conserved regions in StNF-YC4. StNF-YC4 had the highest percent similarity with NtNF-YC4 (87.61%) and the least similarity with GmNF-YC4-2 (75.24%), suggesting StNF-YC4 may have a close evolutionary tie with NtNF-YC4 (Figure 5B,C). There were four regions that can be identified as conserved, where one of them consists of more than half of the total length of StNF-YC4 (Figure 5A).

### 2.7. Promoter Region Analysis Demonstrates the Potential Functional Roles of StNF-YC4

**Figure 6:**
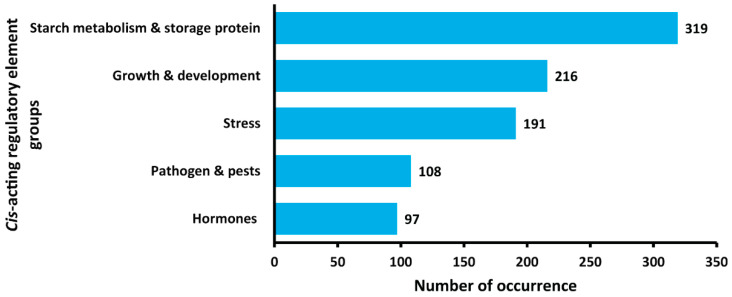
*Cis*-elements in 2000-bp upstream of *StNF-YC4* are grouped into five major categories according to their predicted functions. PlantCARE and PLACE were used to identify these *cis*-elements in *StNF-YC4* promoter regions [48,49].

Analysis of the promoter region (2000-bp sequence upstream of the start codon) of *StNF-YC4* by Nsite-PL (Version 6.2014) discovered five potential functional motifs. Three of them were transcription factor binding sites associated with plants’ uptake and metabolism of carbon and nitrogen (Supplementary Table S2). A regulatory element at −1580 nt upstream was identical to the *Chlamydomonas reinhardtii Nia1* gene encoding nitrate reductase [40]. Another AT-rich regulatory region found in *Lycopersicon esculentum rbcS1* gene encoding Ribulose-1,5-bisphosphate carboxylase-oxygenase (RuBisCO) [41], was identified at −224 nt. Binding site GATA-1, involved in light regulation of nuclear genes encoding chloroplast glyceraldehyde-3-phosphate dehydrogenase in Arabidopsis [42], was present at −304 nt. Together, these findings implicate that *StNF-YC4* may be involved in carbon and nitrogen allocation in potatoes. Furthermore, the binding site Alfin1, associated with salinity tolerance, was recognized at −654 nt, and AGAMOUS, associated with cell maintenance and differentiation in the Arabidopsis floral meristem [43,44], was identified at −958 nt upstream of the start codon of *StNF-YC4*. In addition, PlantCARE and PLACE, two *cis*-element analysis tools, further obtained *cis*-elements in the 2000-bp upstream region of *StNF-YC4,* which were closely associated with starch metabolism, storage proteins, stress response, pests, and pathogen resistance (Figure 6, Supplementary Table S3). Additionally, numerous other *cis*-elements were found in *StNF-YC4* promoter regions associated with plant growth, development, and hormones (Supplementary Table S3).

### 2.8. Analysis of Protein–Protein Interaction Prediction Indicates StNF-YC4’s Possible Role in Stress Resistance and Flowering Time

The potential protein–protein interactions of StNF-YC4 determined by STRING Database identified several proteins that are components of the transcription factor NF-Y complex and are associated with DNA repair and replication (Supplementary Table S4). In addition, there were three components (out of 12) of a network that are associated with salt tolerance, and another three components (out of 16) are associated with flowering time [45,46].

## 3. Discussion

Past studies showed that *QQS* could increase protein content in leaves and seeds of plants such as Arabidopsis, soybean, rice, corn, and tobacco [13,14,15,17,18]. Our protein quantification data here demonstrated a significant increase in tuber protein content when *QQS* was expressed, suggesting *QQS* can boost protein accumulation in the modified stem in potato tuber. This indicates that the function of an orphan may not be limited to a specific species or a particular organ but can be universal among different plants and organs. Moreover, we have also found an increased protein content in potato tubers that are overexpressing *StNF-YC4.* This was consistent with our previous studies, where *NF-YC4* overexpression resulted in similar increases in protein in seeds and leaves of Arabidopsis, soybean, corn, and tobacco [14,16,17,18]. In addition, we observed an overall increase in total protein for both *AtQQS-E* and *StNF-YC4-OE*, and did not notice an increase in a specific protein or a group of proteins (Figure 4C). All mutants expressing *QQS* or overexpressing *StNF-YC4* had reduced starch content.

Publicly available expression data showed that *StNF-YC4* was highly expressed in all organs of potato plants implying their considerable functional role in potatoes [38]. Surprisingly, some known genes involved in carbon and nitrogen allocation, such as *RuBisCO* (Ribulose-1,5-bisphosphate carboxylase-oxygenase, AT1G67090, St homolog PGSC0003DMG400012666), *SWEET10* (Sugar Will Eventually be Exported Transporter 10, AT5G50790, St homolog PGSC0003DMG400031742), *NLP7* (NIN-like protein 7, AT4G24020, St homolog PGSC0003DMG402012256), *NRT1* (Nitrate transporter 1, AT3G16180, St homolog PGSC0003DMG400031742), and *FLOWERING LOCUS T* (FT, AT1G65480, St homolog PGSC0003DMG400023365) have a very low or no expression (~0-5 FPKM) in potato tubers compared to *StNF-YC4* (~70 FPKM) despite being highly expressed in leaves [17,37,38]. Patatin, one of potatoes’ major tuber storage proteins, is associated with FT- and NF-YC-mediated flowering in plants [24,50,51]. It is possible that *StNF-YC4* overexpression may increase the protein content through patatin. However, our SDS-PAGE did not show any noticeable change for a specific protein at the molecular mass of ~45 kDa (Figure 4C), and it is therefore unlikely that the enhanced protein observed is due to the increase of storage protein patatin.

Promoter analysis of *StNF-YC4* identified several transcription factor binding sites involved in carbon and nitrogen uptake, mobilization, and metabolism (Supplementary Tables S2 and S3). Interestingly, a binding site for a transcription factor, ALFIN1, associated with saline resistance in Alfalfa, was also recognized upstream of *StNF-YC4* [43]. In addition, numerous other *cis*-acting elements within 2000-bp upstream of *StNF-YC4* are associated with starch metabolism, storage protein, stress, pathogen, and pest resistance (Supplementary Table S3). Together with the expression profile, these data suggest *StNF-YC4* may be crucial for plants’ carbon and nitrogen allocation, metabolism, defense, growth, and development. Protein–protein interaction prediction analysis also suggested StNF-YC4’s potential function in plants’ stress and flowering time [45,46]. The relationship between *FLOWERING LOCUS T* (*FT*) and NF-Y complex has been thoroughly investigated in the past, and it is understood that *FT* expression is also associated with nitrogen metabolism and storage proteins [24,50,52]. A recent article provided a detailed model explaining how the NF-Y complex may interact with FT to regulate flowering time [53]. However, further studies are necessary to understand the specific role of NF-YC4 in this model. Phenotypic analysis will be key to confirm *StNF-YC4*′s role in stress, pest and pathogen resistance, and flowering time in potato plants. Another recent article reported that *StNF-YC4* (PGSC0003DMT400039470, represented as *StNF-YC1.1* in [54]) was upregulated under Abscisic acid (ABA), drought, and salt stresses, and indicated that *NF-Y* was involved in the regulation of potato growth, development, and response to biotic and abiotic stresses [54]. These findings, together with our starch and protein quantification data, further strengthened our claims that *StNF-YC4* can be a key element of carbon and nitrogen allocation, metabolism, and plants’ response to stress. Sequence alignment outlined multiple conserved regions among different homologs of NF-YC4. Any of these regions, independently or together, might play a crucial role in NF-YC4’s metabolic influence in plants associated with increased protein accumulation.

Growing preference for plant-based protein over animal-based protein has accelerated the search for ways to improve plant protein content. Tuber protein, quality, and yield vary among different cultivars and depend upon various genetic, biotic, abiotic, and environmental factors. Nitrogen availability is one of the key factors that determine tuber protein content in potatoes [55,56,57]. Other components that can alter tuber proteins are growth conditions such as temperature, irrigation, altitude, crop rotation, use of biostimulators, and herbicide [55,56,57,58,59]. This study reports new elements: *QQS*, an orphan gene, and its interactor, *NF-YC4*, that increase tuber protein content without significantly compromising tuber yield. As potato is a popular and highly consumed crop, a high protein potato variety is desired to combat protein deficiency threatening underprivileged populations worldwide. Further study is required to determine *NF-YC4* and *QQS*’ mechanisms and functional basis to successfully integrate this technique for agricultural benefits. Our research may provide a sustainable solution to this issue and can be a model for utilizing plant orphan genes in developing crops with desirable traits.

## 4. Conclusions

Here, we have metabolically analyzed independent *AtQQS-E* and independent *StNF-YC4-OE* potato lines and observed a significant increase in tuber protein and a significant decrease in the tuber starch content compared to the WT potatoes. In addition, no significant change was observed in tuber yield, number, and appearance in potatoes expressing *AtQQS* or overexpressing *StNF-YC4*, suggesting both possess an enormous potential in crop improvement, focusing on obtaining high-protein potatoes. *StNF-YC4* is highly expressed in all potato organs, and several bioinformatics findings described in our study associate it with potato storage protein, metabolism, stress resistance, and defense against pests and pathogens, further reinforcing its promise in crop improvement.

This study demonstrates excellent potential for plant orphan genes and associated interactors in developing high-protein tuber crops. The increased protein from *QQS* and *NF-YC4* shows promise for developing crops with high-protein traits without compromising yield.

## 5. Materials and Methods

### 5.1. Plant Material

We obtained five potato lines expressing *AtQQS* and six potato lines overexpressing *StNF-YC4.* The *AtQQS-E* potato lines were developed by introducing the *AtQQS* coding sequence (CDS) under the control of the cauliflower mosaic virus (CaMV) 35S promoter using the pB2GW7 vector [15] into the ATL variety of potato plants. An ATL potato line with an empty pB2GW7 vector (without *AtQQS* CDS) was also obtained and used as a control for this study (EmptyVector (ATL)). Transgenic *AtQQS-E* plants were identified by the herbicide glufosinate ammonium resistance.

AtNF-YC4 protein sequence was used to search against the potato genome database, and PGSC0003DMP400026764 protein (230 aa, the product of PGSC0003DMT400039470 transcript or PGSC0003DMG402015259 gene transcript) was identified as AtNF-YC4’s homolog in potato. A pCAMBIA-based binary vector was designed to overexpress *StNF-YC4* CDS under the transcriptional control of an enhanced CaMV 35S promoter (2X 35S) in the T-DNA region (Supplementary Figure S1). The T-DNA region of the vector also contained a modified version (W^563^ to L^563^ and S^642^ to I^642^) of the potato *acetolactate synthase* (*mStALS*) gene intended to express under the control of endogenous *Ubiquitin7* promoter (Ubi7) for selection (Supplementary Figure S1) [60,61]. The construct was introduced in the potato variety CWR, known for its high protein content in tubers [62]. An empty vector containing *mStALS* but without *StNF-YC4* CDS was used as a control (EmptyVector (CWR), Supplementary Figure S1). Transgenic *StNF-YC4-OE* plants were selected by herbicide resistance from Imazamox. All tubers used for this study were stored, utilized, and discarded according to the USDA (United States Department of Agriculture) protocol that applies.

### 5.2. Growth Conditions

Each plant line was propagated clonally in growth chambers (with 24 °C temperature, 16 h light/8 h dark photoperiod, light intensity: 80 to 110 μmol photons m^−2^ s^−1^, and ambient humidity ranging from 25 to 50%) by subculturing stem cuttings in a propagation medium (MS-based media, pH 5.7, contains 5.55 g/L Murashige & Skoog Modified Basal Medium with Gamborg Vitamins, PhytoTech Labs, Lenexa, KS, USA), 30 g/L sucrose, 2 g/L Gelzan (Caisson, Smithfield, UT, USA), 300 mg/L Timentin, 1.2 mL/L Plant Preservative Mixture (PPM), and poured into Magenta GA7 vessels for two to four weeks.

Seedlings were later transferred to a greenhouse (with 13 °C night/18 °C day temperature, 16 h light/8 h dark photoperiod, light intensity: 250 to 300 μmol photons·m^−2^·s^−1^, and ambient humidity ranging from 25 to 50%) and grown in 4 L containers in the soil (Sunshine^®^ Mix #1, composed of peat and perlite media with starter fertilizer, Sun Gro^®^ Horticulture, Agawam, MA, USA). Plants were irrigated daily using a drip system in proportion to the growth stage of the plant and fertilized at two, four, and six weeks after planting with Peters Professional Peat Lite Special 20-10-20 (at each application, 240 mL of fertilizer solution was used at a concentration of 840 ppm nitrogen, JR Peters Inc., Allentown, PA, USA). Tubers were harvested three months after planting for all experiments in this study.

### 5.3. Starch Quantification

A modified version using Megazyme’s D-Glucose Assay Kit (GOPOD Format, Megazyme, Wicklow, Ireland) was used to quantify the amount of starch [13]. The potato tubers were peeled, and the tuber tissues were boiled in 80% ethanol. Boiled tissues were then ground with mortars and pestles. After a second 80% ethanol application, samples were boiled in sterilized water. Starch was digested to glucose by α-amylase and amyloglucosidase enzymes. By quantifying the amount of glucose in plant tissue, we determined the starch content using a simple conversion factor as previously described [13].

### 5.4. Protein Quantification and SDS-PAGE

A modified version of the Lowry protein quantification method was used to quantify total protein in the tuber tissues [63]. The peeled tuber tissues were ground in liquid nitrogen and dissolved in a grinding buffer in the presence of protease inhibitors (4-aminobenzoic acid and phenylmethylsulfonyl fluoride) to prevent degradations from proteases. The protein content was determined using a colorimetric chemical reaction technique with Thermo Scientific’s Pierce™ Modified Lowry Protein Assay Kit (Thermo Fisher Scientific Inc., Waltham, MA, USA) [64]. SDS-PAGE was performed. Total protein extracted from potato tuber tissue, as described above, was subjected to a 15% SDS-PAGE separating gel. Coomassie blue (R-250) staining was used to visualize separated proteins.

### 5.5. RT-qPCR

For expression analysis in the tuber, mRNA extracted from tuber tissue was reverse transcribed into cDNA using M-Mulv reverse transcriptase enzymes (New England Biolabs, Ipswich, MA, USA). Resulted cDNA concentration was determined using nanodrop techniques (Thermo Fisher Scientific Inc., Waltham, MA, USA), and an equal amount of cDNA was used to perform RT-qPCR (Applied Biosystems, Waltham, MA, USA). *AtQQS* primers (F: 5′-ATGAAGACCAATAGAGAGCAGGA-3′, R: 5′-TTTTGAGCCTTGCGACACCTGATGT-3′), *StNF-YC4* primers (F: 5′-GACCTACCAACGCCAGGAAA-3′, R: 5′-GGTGCTTCAGCGGAGATCAT-3′), and *Elongation Factor 1-α* (*EF-1α*) gene (PGSC0003DMG400023270; primers, F: 5’-ATTGGAAACGGATATGCTCCA-3′, R: 5′-TCCTTACCTGAACGCCTGTCA-3’) was used as a housekeeping gene to determine the relative transcript levels [65]. Relative expression was determined using the 2^−ΔΔCt^ method [19].

Additionally, to confirm ectopic expression of *AtQQS*, transcript level was also determined in the leaves of the tissue culture (Supplementary Figure S2) using a different set of *AtQQS* primers (F: 5′-TGAAGACCAATAGAGAGCAGGA-3′, R: 5′-GACCCTCATTTTGAGCCTTG-3′) and reference gene: *Adenine Phosphoribosyl Transferase* (*APRT*) gene (NCBI accession number CK270447; primers, F: 5′-GAACCGGAGCAGGTGAAGAA-3′, R: 5′-GAAGCAATCCCAGCGATACG-3′) [65].

### 5.6. Cis-Acting DNA Element Analysis of the Upstream Region of StNF-YC4

The *cis*-acting DNA element analysis was performed (2-kb upstream of the start codon of StNF-YC4) using the Nsite-PL [66,67] (Recognition of PLANT Regulatory motifs with statistics, RegsitePL DB, http://www.softberry.com/berry.phtml?topic=nsitep&group=programs&subgroup=promoter, accessed on 21 September 2021), PlantCARE [49] (a database of plant cis-acting regulatory elements, http://bioinformatics.psb.ugent.be/webtools/plantcare/html/, accessed on 21 September 2021), and PLACE [48] (a Database of Plant *cis*-acting Regulatory DNA elements, https://www.dna.affrc.go.jp/PLACE/?action=newplace, accessed on 21 September 2021).

### 5.7. Multiple Sequence Alignment

Clustal Omega (1.2.4) multiple sequence alignment was used to visualize the alignment of NF-YC4 proteins from different species, and Clustal (2.1) was used to determine percent similarity (https://www.ebi.ac.uk/Tools/msa/clustalo/ accessed on 14 July 2021) [47].

### 5.8. Expression Data

Publicly available expression data were collected from the Potato eFP Browser (http://bar.utoronto.ca/efp_potato/cgi-bin/efpWeb.cgi accessed on 27 February 2022) [37,38]. All expression values are shown as RNA-Seq expression signal in FPKM.

### 5.9. Protein-Protein Interaction

Protein–protein interaction analysis was performed through the STRING Database (https://string-db.org/, accessed on 7 December 2021) [68] to explore protein or networks of protein interactions with StNF-YC4.

### 5.10. Accession Numbers

Sequence information relevant to this study can be obtained under the following accession numbers in the accompanying sources: *AtQQS* (At3g30720) and *AtNF-YC4* (At5g63470.1) coding sequence and peptide sequence were found at TAIR (https://www.arabidopsis.org/, accessed on 9 June 2021). Sequences for GmNF-YC4-1 (Glyma06g17780) and GmNF-YC4-2 (Glyma04g196200) proteins were found at SoyBase (https://www.soybase.org/, accessed on 5 March 2021). *NtNF-YC4* coding and peptide sequences were found at Zhengzhou Tobacco Research Institute of CNTC (Ntab0667000) and at NCBI (AII20181, https://www.ncbi.nlm.nih.gov/, accessed on 9 June 2021). *StNF-YC4* (PGSC0003DMP400026764 peptide, PGSC0003DMT400039470 transcript) were found at International Potato Genome Sequencing Consortium (PGSC) and NCBI (XP_006351509.1, https://www.ncbi.nlm.nih.gov/, accessed on 14 July 2021). For the complete coding sequence of *AtQQS*, *StNF-YC4*, and the peptide sequence of AtNF-YC4, StNF-YC4, NtNF-YC4, GmNF-YC4-1, and GmNF-YC4-2, see Supplementary Table S1.

## Data Availability

The datasets presented in this study can be found in online repositories. The names of the repository/repositories and accession number(s) can be found in the article/Supplementary Material.

## Supplementary Materials

The following supporting information can be downloaded at: https://www.mdpi.com/article/10.3390/plants11223076/s1, Figure S1: The *StNF-YC4-OE* construct was used to transform the potato (*Solanum tuberosum*, Clearwater variety); Figure S2: Relative expression of *AtQQS* transcript in the potato leaf of *AtQQS-E* lines; Figure S3: *StNF-YC4* (PGSC0003DMP400026764) expression in different organs of potato plants from publicly available data (electronic fluorescent pictograph); Figure S4: *StNF-YC4* (PGSC0003DMP400026764) expression signal (FPKM) in different organs of potato from publicly available RNA-Seq data; Table S1: The coding sequence of *AtQQS* and *StNF-YC4*, and the peptide sequence of AtNF-YC4, StNF-YC4, NtNF-YC4, GmNF-YC4-1, and GmNF-YC4-2; Table S2: Transcription factor binding sites are predicted in the *StNF-YC4* promoter region (2-kb upstream of the StNF-YC4 start codon) by Nsite-PL; Table S3: Putative cis-acting DNA elements in the promoter region (2-kb upstream of the StNF-YC4 start codon) of the *StNF-YC4* gene analyzed by PlantCARE and PLACE; Table S4: Protein–protein interactions of StNF-YC4 are predicted by STRING Database. Refs[37,38,40,41,42,43,44,48,49,66,67,68] are citied in supplementary materials
