# Supplementary material for: Orphan Genes in Crop Improvement: Enhancing Potato Tuber Protein without Impacting Yield
Source: Plants (Basel). 2022 Nov 13;11(22):3076. doi: 10.3390/plants11223076 (PMC9696052; doi:10.3390/plants11223076)
Supplement: Supplementary file 1 [file plants-11-03076-s001.zip › plants-1984663-supplementary.pdf]

plants

Supplementary Materials

for

Orphan Genes in Crop Improvement: Enhancing Potato Tuber Protein without Impacting Yield

Rezwan Tanvir [1], Lei Wang [1], Amy Zhang [1,2], Ling Li [1,*]

[1] Department of Biological Sciences, Mississippi State University, Starkville, MS, 39762, USA
[2] Mississippi School for Mathematics and Science, Columbus, MS, 39701, USA
* **Correspondence:** liling@biology.msstate.edu; Tel.: +1-662-325-7570

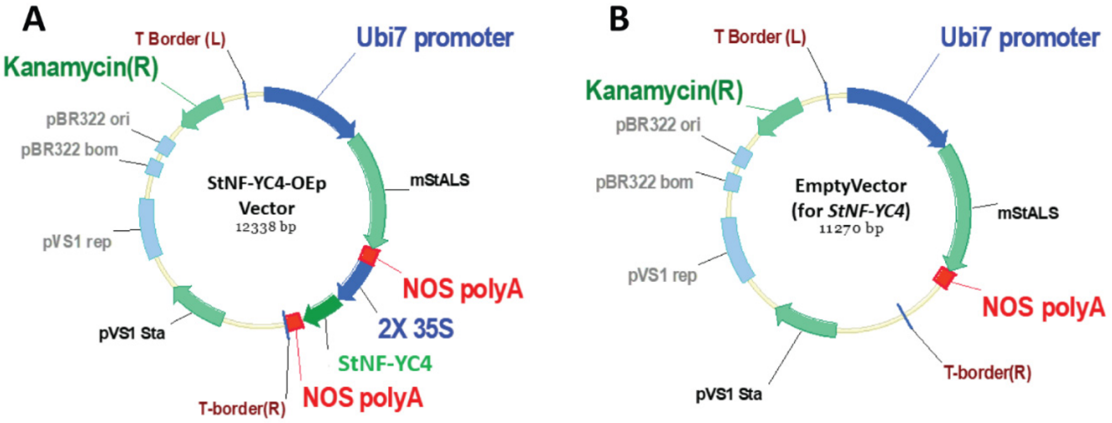

Supplementary Figure S1. The StNF-YC4-OE construct was used to transform the potato (*Solanum tuberosum*, Clearwater variety). (**A**) The complete *StNF-YC4* coding sequence (CDS) from *S. tuberosum* was inserted into a pCAMBIA-based vector under the control of an enhanced CaMV 35S promoter (2X 35S) and nopaline synthase (NOS) terminator. The construct also contained a modified version of potato *acetolactate synthase* (*StALS*, PGSC0003DMG400034102) under the control of *Ubiquitin7* promoter (Ubi7) and a NOS terminator, providing resistance against the ALS inhibitors for selection. (**B**) The empty vector without the *StNF-YC4* CDS, 2X 35S promoter, and C-Terminal NOS terminator was used as a control.

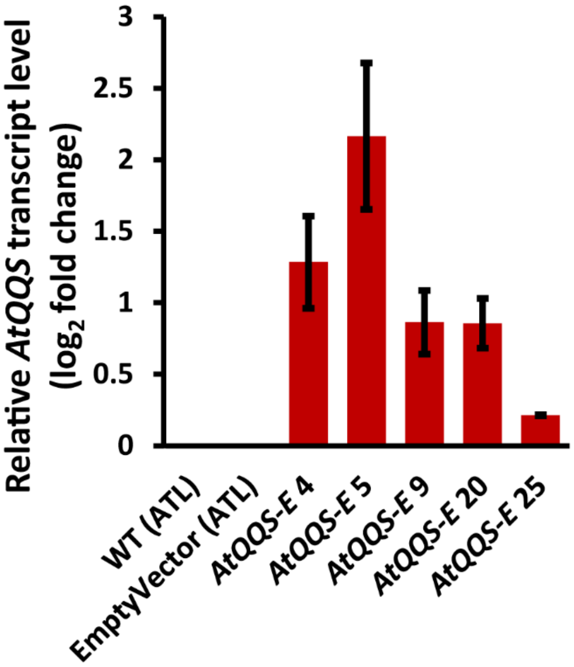

Supplementary Figure S2. Relative expression of *AtQQS* transcript in the potato leaf of *AtQQS-E* lines. The relative transcript levels of *AtQQS* were quantified by RT-qPCR using the *adenine phosphoribosyltransferase* (*APRT*) gene as a reference. The transcript level values are transformed by log_2 fold change. All tubers are of ATL variety.

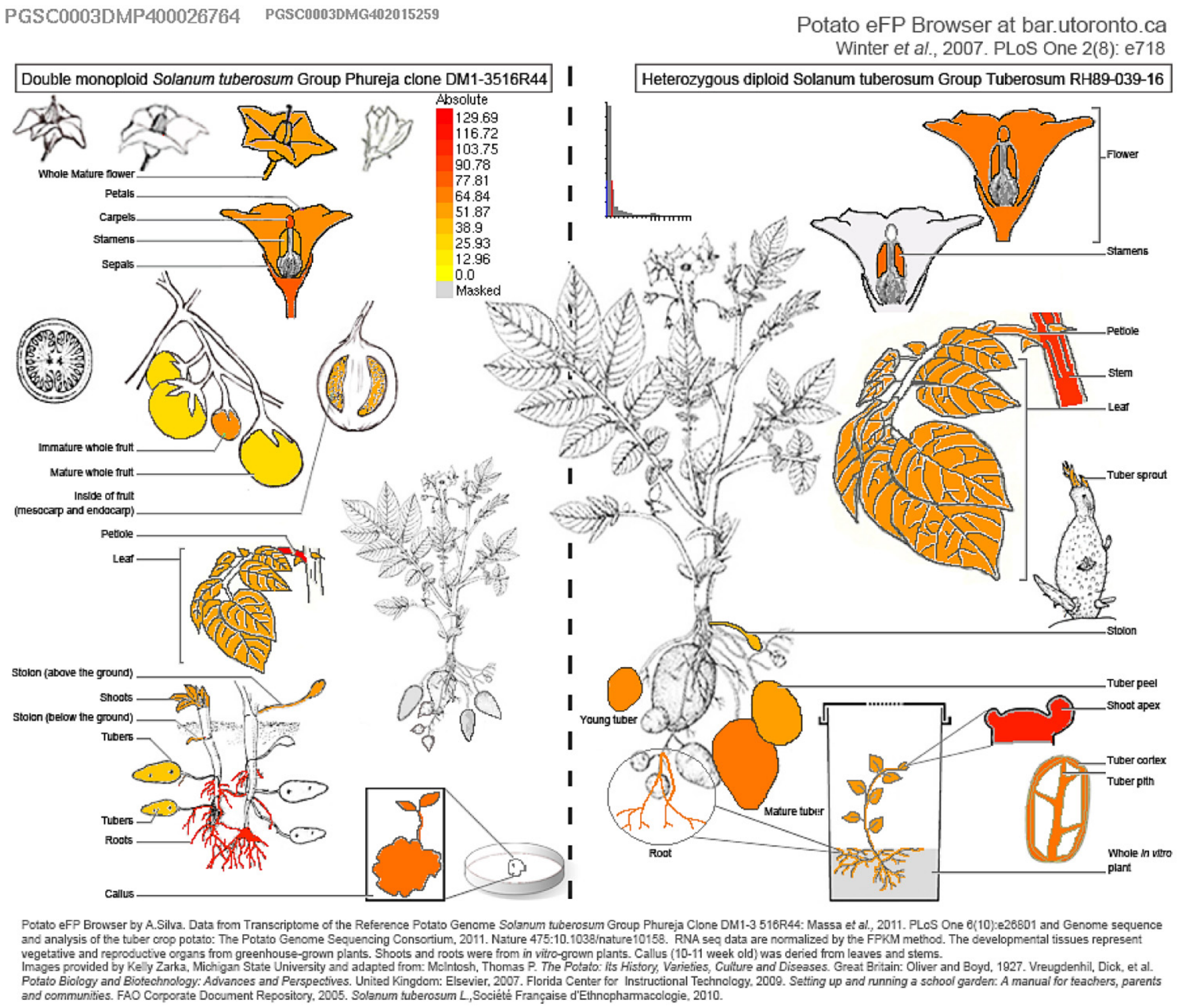

Supplementary Figure S3. *StNF-YC4* (PGSC0003DMP400026764) expression in different organs of potato plants from publicly available data (electronic fluorescent pictograph) (http://bar.utoronto.ca/efp_potato/cgi-bin/efpWeb.cgi) [1,2].

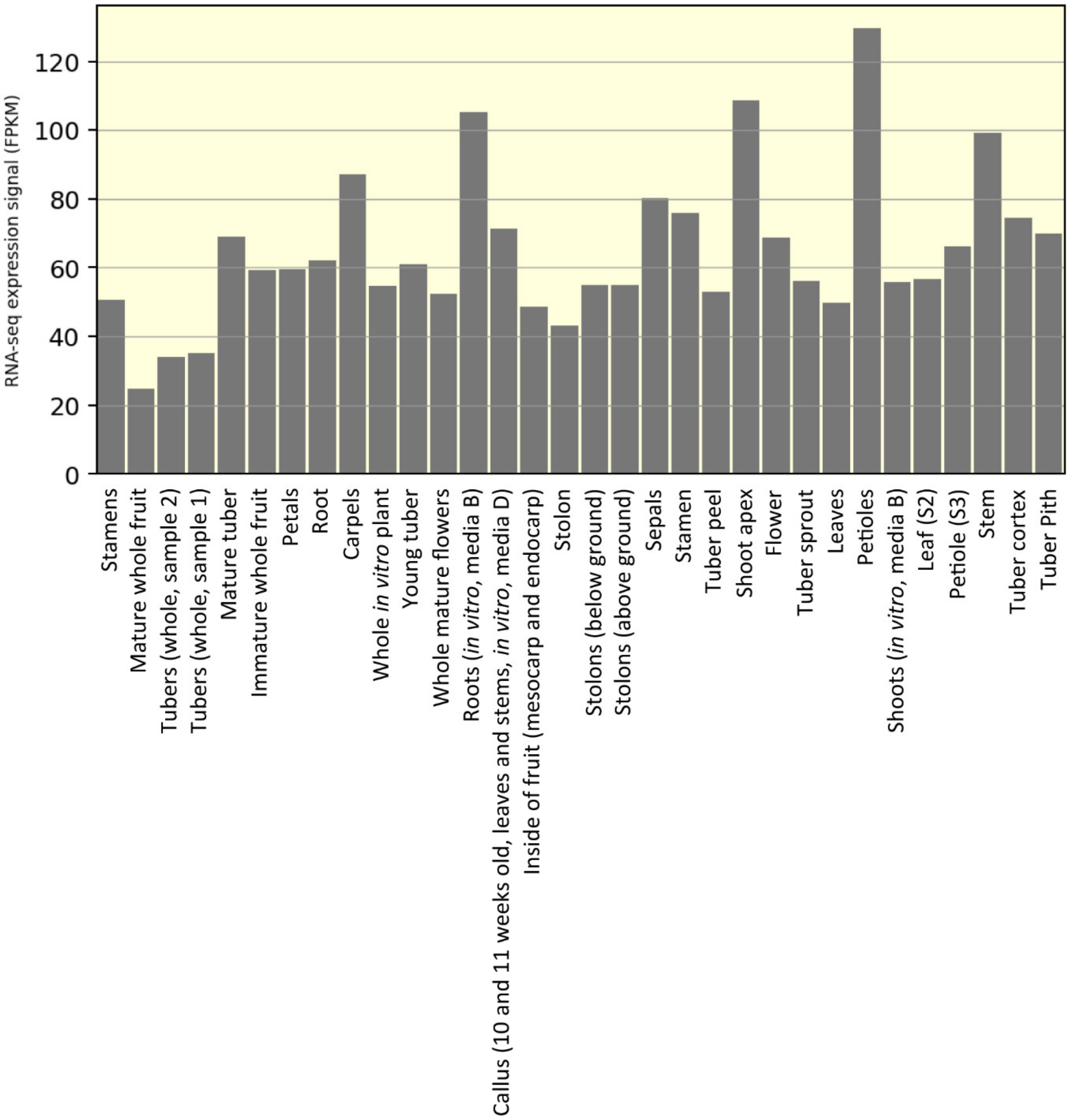

Supplementary Figure S4. *StNF-YC4* (PGSC0003DMP400026764) expression signal (FPKM) in different organs of potato from publicly available RNA-Seq data (http://bar.utoronto.ca/efp_potato/cgi-bin/efpWeb.cgi) [1,2].

Supplementary Table S1. The coding sequence of *AtQQS* and *StNF-YC4* and the peptide sequence of AtNF-YC4, StNF-YC4, NtNF-YC4, GmNF-YC4-1, and GmNF-YC4-2. The start and stop codon are underlined. The AtNF-YC4 top homolog in the potato genome was StNF-YC4 (PGSC0003DMP400026764 peptide, PGSC0003DMT400039470 transcript, NCBI ref. XP_006351509.1). Its sequence was obtained from the International Potato Genome Sequencing Consortium (PGSC) (access date 14 July 2021). *AtQQS* (At3g30720) and *AtNF-YC4* (At5g63470) coding and peptide sequences were found in TAIR (https://www.arabidopsis.org/) (access date 09 June 2021). Sequences for GmNF-YC4-1 (Glyma06g17780) and GmNF-YC4-2 (Glyma04g196200) proteins were found in SoyBase (https://www.soybase.org/) (access date 05 March 2021). NtNF-YC4 peptide sequences were found at Zhengzhou Tobacco Research Institute of CNTC (Ntab0667000) and NCBI (AII20181, https://www.ncbi.nlm.nih.gov/).

Gene	Sequence information
AtQQS<br>Coding<br>sequence	<u>ATG</u>AAGACCAATAGAGAGCAGGAAATTTACGTTGAAAGAAGCTTCAAAC-<br>CAAACAATTCAACAATTCAGAATTTGATGGACATTGAAAGGTTCATTTTGCCTCACACTTCTACATCA<br>GGTGTCGCAAGGCTCAAAATGAGGGTCATATCATGGGTCGGGCTTCAGTTCTACAACTAC<u>TGA</u>
StNF-YC4<br>Coding<br>sequence	<u>ATG</u>GAAAACAGCCCTCAGCAATCGGCGGCGAATGCAGCGGCGGCAGCTGCAGCAGCTCAG-<br>TCCGCTGGGTATCCGACTCAGCCACCTTACCACCATCTCCTTCAGCAACAGCAGCAGCAGTTACAGAT<br>GTTTTGGACCTACCAACGCCAGGAAATCGAACAGGTTAACGATTTTAAGAAC-<br>CATCAACTTCCCCTTGCCCGAATCAAGAAGATCATGAAAGCTGACGAAGATGTCCGTATGATCTCCG<br>CTGAAGCACCCATTTTGTTTGCCAAGGCATGTGAGCTTTTCATTCTGGAACTCAC-<br>TATTCGTTCCTGGCTTCACGCTGAGGAAAACAAGCGACGGACTCTGCAGAAGAATGACATCGCTGCG<br>GCGATTACGCGGACTGACATTTTCGATTTCCTTGTTGACATTGTTCCGAGGGATGA-<br>GATCAAGGAAGAGGATGCTGGTCTTGGGCCTGGCATGGTGGGTTCCACAGCAAGTGGTGTTCCGTAC<br>TATTATCCACCAATGGGACAGCCTGCTCCACCTGGGGTGATGATGGGAAGGCCTGC-<br>TATGCCTGGGGTTGATCCTTCAATGTACGTCCAGCCTCCGCCACCACCATCGCAGGCGTGGCAGTCCG<br>TTTGGCAGGCTGCTGAGGATAATTCTTATGCTGGTGGAGGCAGCAATGGACAGGGTAACCTT-<br>GATGGTCAAAGG<u>TAA</u>
AtNF-YC4<br>Peptide<br>sequence	MDNNNNNNNQQPPPTSVYPPGSAVTTVIPP PPSGSASIVTGGGATYHHLLQQQQQLQMFWTYQRQEI-<br>EQVNDFKNHQLPLARIKKIM-<br>KADEDVRMISAEAPILFAKACELFILELTIRSWLHAEENKRRTLQKNDIAAAITRTDIFDFLVDIVPREEIKEEE<br>DAASALGGGGMVAPAASGVPYYYPPMGQPAVPGGMMIGRPAMDPSGVYAQPP-<br>SQAWQSVWQNSAGGGDDVSYGSGGSSGHGNLDSQG
StNF-YC4<br>Peptide<br>sequence	MENSPQQSAANAAAAAAAAQSAGYPTQPPYHHLLQQQQQLQMFWTYQRQEI-<br>EQVNDFKNHQLPLARIKKIMKADEDVRMISAEAPILFAKACELFILELTIRSWLHAEENKRRTLQKNDIAAAI<br>TRTDIFDFLVDIVPRDEIKEEDAGLGPGMVGSTASGVPYYYPPMGQPAPPGVMMGRPAM-<br>PGVDPSMYVQPPPPPSQAWQSVWQAAEDNSYAGGGSNGQGNLDGQS*
NtNF-YC4<br>Peptide<br>sequence	MDNNPHQSPTTAAAAAAAAQSAPYPQQTPYHHLLQQQQQLQMFWTYQRQEI-<br>EQANDFKNHQLPLARIKKIMKADEDVRMISAEAPILFAKACELFILELTIRSWLHAEENKRRTLQKNDIAAAI<br>TRTDIFDFLVDIVPRDEIKDESGVVGLGPGMVGPTASGVPYYYPPLGQPTPPGGVMMGRPAP-<br>MAGVDPAAFYSQPPSQAWQWQTTEDNSYASGGSSGQGNLDGQK
GmNF-YC4-1<br>Peptide<br>sequence	METNNQQQQQGAQAQSGPYPVAGAGGSAGAGAGAPPPFQHLLQQQQQLQMFWSYQRQEI-<br>EHVNDFKNHQLPLARIKKIMKADEDVRMISAEAPILFAKACELFILELTIRSWLHAEENKRRTLQKNDIAAAI<br>TRTDIFDFLVDIVPRDEIKDDAALVGATASGVPYYYPPIGQPAGMMIGRPAV-<br>DPATGVYVQPPSQAWQSVWQSAAEDASYGTGGAGAQRSLDGQS
GmNF-YC4-2<br>Peptide<br>sequence	MENNQQQGAQAQSGPYPGGAGGSAGAGAGAGAAPFQHLLQQQQQLQMFWSYQRQEI-<br>EHVNDFKNHQLPLARIKKIMKADEDVRMISAEAPILFAKACELFILELTIRSWLHADENKRRTLQKNDIAAA<br>ITRTDIFDFLVDIVPRDEIKDDAALVGATASGVPYYYPPIGQPAGMMIGRPAV-<br>DPATGVYVQPPSQAWQSVWQSAAEDTPYGTGAQGNLDGQSMEF

Supplementary Table S2. Transcription factor binding sites are predicted in the *StNF-YC4* promoter region (2-kb upstream of the StNF-YC4 start codon) by Nsite-PL [3,4].

Position	TF binding site	Verified potato TF	Function
-224	TCAAATATATtA AAATaTaaT	Unknown nuclear factor	AT-rich regulatory region found at *Lycopersicon esculentum rbcS1* gene [5]
-304	TATCCCCAATTT AATC	GATA-1	Involved in light regulation of nuclear genes encoding chloroplast glyceraldehyde-3-phosphate dehydrogenase in *Arabidopsis* [6]
-654	AAAGTGGGGcCA	Alfin1	Alfalfa gene improves salinity tolerance of the plants. Alfin1 expression is essential for normal plant development [7]
-958	AGTGActaA-TAGGTAA	AGAMOUS	Induces the expression of KNU, A timing mechanism for stem cell maintenance and differentiation in the *Arabidopsis* floral meristem [8]
-1580	GgAGGGTTCAAA	Unknown nuclear factor, ARE 76/65	Regulatory elements found at *Chlamydomonas reinhardtii Nia1* gene encoding nitrate reductase [9]

Supplementary Table S3. Putative *cis*-acting DNA elements in the promoter region (2-kb upstream of the StNF-YC4 start codon) of the *StNF-YC4* gene analyzed by PlantCARE and PLACE [10,11].

Key Elements Name	*Cis*-Acting Regulatory Elements	Occurrence	Putative Function	Functional Category
-10PEHVPSBD	TATTCT	4	-10 promoter element found in the barley chloroplast *psbD* gene promoter; involved in light regulation, photosynthesis, circadian system	Growth and development, starch metabolism
AAGAA-motif	GAAAGA, gGTAAAGAAA	2	A motif found at the promoter region of plant *β-galactosidase* (*β-Gal*), *glycosyl hydrolase*; associated with β-galactosidase, cell wall biogenesis, and fruit softening.	Starch metabolism and storage protein
ACGTATERD1	ACGT	2	ACGT sequence required for expression of *erd1* (*early responsive to dehydration*) in *Arabidopsis*	Abiotic stress
AMYBOX1, AMYBOX2	TAACARA, TATCCAT	4	Conserved sequence found in the upstream region of *α-amylase* genes	Starch metabolism and storage protein
ANAERO1CONSENSUS, ANAERO2CONSENSUS, ANAERO3CONSENSUS	AAACAAA, AGCAGC, TCATCAC	4	Part of 16 motifs found in silico in promoters of 13 anaerobic genes involved in the fermentative pathway; associated with oxygen stress, Kreb's cycle, glycolysis	Abiotic stress, starch metabolism, and storage protein
ARFAT	TGTCTC	2	Auxin response factor binding site found in the promoters of primary/early auxin response genes	Hormones, growth, and development
ARR1AT	NGATT	19	ARR1-binding element found in *Arabidopsis*; associated with bacterial and environmental stimuli response regulators	Pathogen and pest resistance; Abiotic stress
ASF1MOTIFCAMV	TGACG	1	ASF-1 binding site; associated with auxin, SA, xenobiotic stress, and disease resistance	Pathogen, pest, abiotic stress; plant hormones
BIHD1OS	TGTCA	6	The binding site of a rice TF, BELL; associated with disease resistance responses	Pathogen and pest resistance
Box 4	ATTAAT	5	Part of a conserved DNA module involved in light responsiveness, photosynthesis	Starch metabolism and storage protein
Box S	AGCCACC	1	Promoter motifs involved in the network of Phytochrome A-regulated gene expression	Starch metabolism and storage protein
CACTFTPPCA1	YACT	31	Key component of Mem1 in the cis-regulatory element of the phosphoenolpyruvate carboxylase	Starch metabolism and storage protein
CAREOSREP1	CAACTC	1	RE found in the promoter of a *cysteine proteinase*; associated with GA, aleurone, storage protein	Hormones, growth and development, and storage protein
CARGCW8GAT	CWWWWWWWWG	6	Binding site for AGL15; associated with maturity, senescence	Growth and development

CAT-box	GCCACT	1	*Cis*-acting RE related to meristem-specific expression	Growth/ development, metabolism
CGTCA-motif	CGTCA	1	*Cis*-acting RE involved in MeJA-responsiveness; associated with drought, abiotic stress, JA	Abiotic stress, hormones
chs-CMA2a	TCACTTGA	1	Part of a light-responsive element; associated with photosynthesis, starch metabolism	Starch metabolism and storage protein
CPBCSPOR	TATTAG	3	Critical for Cytokinin-enhanced protein binding; associated with chloroplast, photosynthesis	Hormones; starch and storage protein
CURECORECR	GTAC	4	Core of a copper-response element; associated with hypoxic or oxygen stress	Abiotic stress
DOFCOREZM	AAAG	32	Core site binding of Dof proteins; associated with carbon metabolism	Starch metabolism and storage protein
DPBFCORED-CDC3	ACACNNG	4	A novel class of bZIP transcription factors binding core sequence, associated with ABA	Hormones; growth and development
EBOXBNNAPA	CANNTG	24	E-box of napA storage-protein gene; associated with, R-response element, and light response	Abiotic stress; Starch and storage protein
EECCRCAH1	GANTTNC	3	Consensus motif of the enhancer elements found in the promoter region of the *Cah1* (encoding carbonic anhydrase); associated with low-CO2	Abiotic stress, starch metabolism, and storage protein
ERE, ERELEE4	ATTTTAAA, AW-TTCAAA	4	Ethylene-responsive element; associated with ethylene, seed germination, root nodulation, flower senescence, metabolism, and fruit ripening	Hormones, growth and development, starch and storage protein
G-Box	GCCAC-GTGGA, TCCACATGGCA	2	*Cis*-acting RE involved in light responsiveness; associated with REs under specific stress	Abiotic stress, growth, and development
GARE1OSREP1, GAREAT	TAACAGA, TAACAAR	3	Gibberellin-responsive element; associated with GA, seed germination, starch hydrolysis	Hormones, starch, growth/ development,
GATABOX	GATA	15	Motif in the promoter of *chlorophyll a/b binding protein*, *Cab22*; associated with photosynthesis	Starch metabolism and storage protein
GT1CONSEN-SUS, GT1CORE, GT1GMSCAM4	GRWAAW, GGTTAA, GAAAAA	43	GT-1 motif and Consensus GT-1 binding site; associated with SA-induced gene expression, RUBISCO, pathogen-, light- and salt-induced expression	Pathogen and pest resistance, abiotic stress, growth and development, starch
GTGANTG10	GTGA	8	Motif in the promoter of late pollen gene; involved in pollen, pectate lyase, reproduction	Growth and development
IBOX, IBOX-CORE	GATAAG, GATAA	6	I-box: Conserved sequence upstream of light-regulated genes; associated with Le-MYB1 (MYB like) proteins, light response, differentiation, RUBISCO, stress, photosynthesis, and defense	Pathogen and pest resistance, abiotic stress, growth and development, starch

LECPLEACS2	TAAAATAT	4	Core element in Cys protease binding *cis*-element, associated with ethylene, xylanase, wound response, seed germination, root hair/nodulation, flower senescence, abscission, and fruit ripening	Abiotic stress, hormones, growth and development, starch and storage protein
MYB, MYB1AT, MYBCORE, MYB-like sequence, MYBPZM, MYBST1	TAACCA, WAACCA, CNGTTR, TAACCA, CCWACC, GGATA	9	MYB or MYB-homolog recognition sites and core or consensus MYB binding sites; associated with plant's defense, stress including dehydration stress, ABA, development, differentiation, flavonoid biosynthesis	Pathogen and pest resistance, abiotic stress, growth and development, hormones, starch metabolism
MYC, MYCATERD1, MYCATRD22, MYCCONSENSUSAT	CAATTG, CATGTG, CACATG, CANNTG	39	MYC *cis*-elements, binding and recognition sites; associated with cold stress, JA, light signaling, dehydration, ABA	Abiotic stress, hormones, growth, and development, starch metabolism
NAPINMOTIFBN	TACACAT	3	Found in upstream region *2S albumin* gene; associated with seed storage protein	Starch metabolism and storage protein
NTBBF1ARROLB	ACTTTA	2	Dof protein from tobacco binding site; associated with auxin, tissue-specific expression	Hormones, growth, and development
O2-site	GATGATGTGG	1	*Cis*-acting RE involved in zein metabolism	Metabolism
OSE2ROOTNODULE	CTCTT	4	Consensus sequence motifs of the promoters activated in infected cells of root nodules	Pathogen and pest resistance
POLASIG1, POLASIG2, POLASIG3	AATAAA, AATTAAA, AATAAT	31	Poly A signal (or consensus sequence) found in the promoter regions of the *α-amylase* gene	Starch metabolism and storage protein
POLLEN1LELAT52	AGAAA	16	Co-dependent REs responsible for pollen-specific activation of *lat52* gene; associated with endo-β-mannanase, pollen-specific expression	Growth and development
PREATPRODH	ACTCAT	1	Pro- or hypoosmolarity-responsive element in the promoter region of *proline dehydrogenase* gene	Abiotic stress
PYRIMIDINEBOXHVEPB1, PYRIMIDINEBOXOSRAMY1A	TTTTTTCC, CCTTTT	3	Found in the *cysteine proteinase* gene promoter; associated with GA, ABA, germination, root hair/ nodulation, senescence, abscission, and fruit ripening, stress and wound, aleurone, protein degradation, *α*-amylase, sugar repression,	Abiotic stress, hormones, growth and development, starch and storage protein
QELEMENTZMZM13	AGGTCA	1	Q element in *ZM13* gene promoter; associated with pollen, hybrid breeding, and seed production	Growth and development
RAV1AAT	CAACA	1	Consensus sequence of TF, RAV1; associated with pathogen-induced transcription, drought and salt tolerance, germination, and seedling development	Pathogen, pest, abiotic stress, growth and development
RBCSCONSENSUS	AATCCAA	1	Consensus sequence for RUBISCO	Growth and development, starch

ROOTMO-TIFTAPOX1	ATATT	32	Motif found in promoters of *rolD*; associated with early reproductive phase and flowering	Growth and development
SEB-FCONSSTPR10A	YTGTCWC	10	Binding site of SEBF pathogenesis-related gene (*PR-10a*)	Pathogen and pest resistance
SEF1MOTIF, SEF3MO-TIFGM, SEF4MO-TIFGM7S	ATATTTAWW, AACCCA, RTTTTTR	9	Binding motif (or consensus sequence) found in upstream region *β-conglicinin* (7S globulin); associated with legume seed storage protein	Starch metabolism and storage protein
SORLIP1AT, SORLIP2AT, SORLREP3AT	GCCAC, GGGCC, TGTATA-TAT	8	Sequences over-represented in light-induced or repressed, phyA-induced motifs; associated with light-induced and root-specific genes expression	Growth/ development, starch metabolism, and storage protein
SP8BFIBSP8BIB	TACTATT	1	SPBF binding site at the upstream of *sporamin* gene and *β-amylase* gene	Starch metabolism and storage protein
SREATMSD	TTATCC	2	Sugar-repressive element associated with stem decapitation and axillary bud outgrowth	Growth and development
STRE	AGGGG	5	The SEB-1 TF binds to the STRE Motif; associated with regulation of cellular processes, stress response, and reserve carbohydrate metabolism	Abiotic stress, growth, and development, starch metabolism
SURECOREATSULTR11	GAGAC	2	Core of sulfur-responsive element in the promoter of *SULTR1*; associated with sucrose responsive element, auxin, sulfur response, stress	Abiotic stress, hormones, growth, and development, starch
SV40COREEN-HAN	GTGG-WWHG	1	SV40 core enhancer, similar sequences found in *RUBISCO* genes	Starch metabolism
TAAAGSTKST1	TAAAG	10	TAAAG motif in the promoter of *S. tuberosum KST1* gene; associated with expression of multiple genes involved in carbon metabolism	Starch metabolism and storage protein
TATAPVTR-NALEU	TTTATATA	1	A TATA-like motif; associated with carbohydrate degradation	Growth/ development, starch metabolism
TATCCA-OSAMY, TATCCAYMO-TIFOSRAMY3D	TATCCA, TATCCAY	4	Found in *α-amylase* promoters; associated with MYB proteins, GA, plant defense, biotic and abiotic stresses	Pathogen/ pest, abiotic stress, hormones, growth/ development
TCA-element	CCATCTTTTT	1	SA response element; associated with systemic expression of pathogenesis-related proteins	Pathogen and pest resistance
TGACG-motif	TGACG	1	*Cis*-acting regulatory element involved in the MeJA-responsiveness; associated with plant defense and development, JA	Pathogen and pest resistance, hormones, growth/ development
TGAC-GTVMAMY	TGACGT	1	Found in the *α-amylase* gene promoter; associated with expression of *α-amylase* in the cotyledons of the germinated seeds, MYB proteins	Growth/ development, starch metabolism, and storage protein

Unnamed__4	CTCC	11	Sequence within *DYT1* promoter; associated with *DYT1* expression, anther development/ function.	Growth and development
WBOX-ATNPR1, WBOXNT-CHN48, WBOXNTERF3	TTGAC, CTGACY, TGACY	11	W-box found in the promoter *WRKY*; Associated with SA, disease resistance, elicitor-response, wound response, and stress resistance	Pathogen and pest resistance, abiotic stress, hormones
WBOXHVISO1	TGACT	4	W-box element in *isoamylase1* promoter; associated with patatin, WRKY, isoamylase, starch	Starch metabolism and storage protein
WRE3, WUN-motif	CCACCT, CAATTACAT	3	Wound-response element	Abiotic stress
WRKY71OS	TGAC	12	Binding site of WRKY71 at *amylase* promoter; associated with GA and *Pathogenesis-Related Class10* (*PR-10*) genes	Pathogen/ pest, abiotic stress, hormones, starch metabolism
WUSATAg	TTAATGG	1	Target sequence of WUS in the intron of *AGAMOUS* gene	Growth and development

Supplementary Table S4. Protein-protein interactions of StNF-YC4 are predicted by STRING Database [12].

ID	Details	Scores
PGSC0003DMT400067767	Uncharacterized protein; DNA polymerase epsilon P17 subunit	0.967
PGSC0003DMT400010977	Nuclear transcription factor y subunit b-3; Uncharacterized protein; CCAAT-binding transcription factor subunit A	0.950
PGSC0003DMT400004366	Nuclear transcription factor y subunit b-10-like; Uncharacterized protein; CCAAT-binding transcription factor subunit A	0.922
PGSC0003DMT400054416	Uncharacterized protein; Nuclear transcription factor Y subunit A-4	0.893
PGSC0003DMT400079711	DNA polymerase epsilon subunit; Participates in DNA repair and in chromosomal DNA replication	0.876
PGSC0003DMT400055061	Nuclear transcription factor y subunit a-10; Uncharacterized protein; YA1	0.853
PGSC0003DMT400072322	Nuclear transcription factor y subunit a-10-like; Uncharacterized protein; YA1	0.853
PGSC0003DMT400095009	Nuclear transcription factor y subunit b-3-like; Uncharacterized protein; CCAAT-binding transcription factor subunit A	0.852
PGSC0003DMT400000740	Uncharacterized protein; YA5	0.819
PGSC0003DMT400006358	Nuclear transcription factor y subunit a-3-like; Uncharacterized protein; YA6	0.819

References

1. Winter, D.; Vinegar, B.; Nahal, H.; Ammar, R.; Wilson, G.V.; Provart, N.J. An "Electronic Fluorescent Pictograph" browser for exploring and analyzing large-scale biological data sets. *PLoS One* **2007**, *2*, e718. https://doi.org/10.1371/journal.pone.0000718.
2. Massa, A.N.; Childs, K.L.; Lin, H.; Bryan, G.J.; Giuliano, G.; Buell, C.R. The transcriptome of the reference potato genome *Solanum tuberosum* Group Phureja clone DM1-3 516R44. *PLoS One* **2011**, *6*, e26801. https://doi.org/10.1371/journal.pone.0026801.
3. Shahmuradov, I.A.; Solovyev, V.V. Nsite, NsiteH and NsiteM computer tools for studying transcription regulatory elements. *Bioinformatics* **2015**, *31*, 3544-3545. https://doi.org/10.1093/bioinformatics/btv404.
4. Solovyev, V.V.; Shahmuradov, I.A.; Salamov, A.A. Identification of promoter regions and regulatory sites. In *Computational Biology of Transcription Factor Binding*, Ladunga, I., Ed.; Springer: New Jersey, 2010; pp. 57-83.
5. Outchkourov, N.S.; Peters, J.; De Jong, J.; Rademakers, W.; Jongsma, M.A. The promoter–terminator of chrysanthemum *rbcS1* directs very high expression levels in plants. *Planta* **2003**, *216*, 1003-1012. https://doi.org/10.1007/s00425-002-0953-8.
6. Jeong, M.-J.; Shih, M.-C. Interaction of a GATA factor with cis-acting elements involved in light regulation of nuclear genes encoding chloroplast glyceraldehyde-3-phosphate dehydrogenase in Arabidopsis. *Biochem. Biophys. Res. Commun.* **2003**, *300*, 555-562. https://doi.org/10.1016/S0006-291X(02)02892-9.
7. Winicov, I.I.; Bastola, D.R. Transgenic overexpression of the transcription factor *alfin1* enhances expression of the endogenous *MsPRP2* gene in alfalfa and improves salinity tolerance of the plants. *Plant Physiol.* **1999**, *120*, 473-480. https://doi.org/10.1104/pp.120.2.473.
8. Sun, B.; Xu, Y.; Ng, K.-H.; Ito, T. A timing mechanism for stem cell maintenance and differentiation in the *Arabidopsis* floral meristem. *Genes Dev.* **2009**, *23*, 1791-1804. https://doi.org/10.1101/gad.1800409.
9. Loppes, R.; Radoux, M.; Ohresser, M.C.P.; Matagne, R.F. Transcriptional regulation of the *Nia1* gene encoding nitrate reductase in *Chlamydomonas reinhardtii*: effects of various environmental factors on the expression of a reporter gene under the control of the *Nia1* promoter. *Plant Mol. Biol.* **1999**, *41*, 701-711. https://doi.org/10.1023/A:1006381527119.
10. Higo, K.; Ugawa, Y.; Iwamoto, M.; Korenaga, T. Plant cis-acting regulatory DNA elements (PLACE) database: 1999. *Nucleic Acids Res.* **1999**, *27*, 297-300. https://doi.org/10.1093/nar/27.1.297.
11. Lescot, M.; Déhais, P.; Thijs, G.; Marchal, K.; Moreau, Y.; Van de Peer, Y.; Rouzé, P.; Rombauts, S. PlantCARE, a database of plant cis-acting regulatory elements and a portal to tools for in silico analysis of promoter sequences. *Nucleic Acids Res.* **2002**, *30*, 325-327. https://doi.org/10.1093/nar/30.1.325.
12. Szklarczyk, D.; Gable, A.L.; Nastou, K.C.; Lyon, D.; Kirsch, R.; Pyysalo, S.; Doncheva, N.T.; Legeay, M.; Fang, T.; Bork, P.; et al. The STRING database in 2021: customizable protein-protein networks, and functional characterization of user-uploaded gene/measurement sets. *Nucleic Acids Res.* **2021**, *49*, D605-D612. https://doi.org/10.1093/nar/gkaa1074.
